# Improved Biomineralization Using Cellulose Acetate/Magnetic Nanoparticles Composite Membranes

**DOI:** 10.3390/polym17020209

**Published:** 2025-01-15

**Authors:** Madalina Oprea, Andreea Madalina Pandele, Aurelia Cristina Nechifor, Adrian Ionut Nicoara, Iulian Vasile Antoniac, Augustin Semenescu, Stefan Ioan Voicu, Catalin Ionel Enachescu, Anca Maria Fratila

**Affiliations:** 1 Advanced Polymers Materials Group, National University of Science and Technology POLITEHNICA Bucharest, 1-7 Gheorghe Polizu, 011061 Bucharest, Romania; madalinna_09@yahoo.com (M.O.); pandele.m.a@gmail.com (A.M.P.); 2 Department of Analytical Chemistry and Environmental Engineering, Faculty of Chemical Engineering and Biotechnologies, National University of Science and Technology POLITEHNICA Bucharest, 1-7 Gheorghe Polizu, 011061 Bucharest, Romania; aureliacristinanechifor@gmail.com; 3 Department of Science and Engineering of Oxide Materials and Nanomaterials, Faculty of Chemical Engineering and Biotechnologies, National University of Science and Technology POLITEHNICA Bucharest, 1-7 Gheorghe Polizu, 011061 Bucharest, Romania; adrian.nicoara@upb.ro; 4 Faculty of Materials Science and Engineering, National University of Science and Technology POLITEHNICA Bucharest, 313 Splaiul Independentei, 060042 Bucharest, Romania; iulian.antoniac@upb.ro (I.V.A.); augustin.semenescu@upb.ro (A.S.); 5 Academy of Romanian Scientists, 54 Splaiul Independentei Street, District 5, 050094 Bucharest, Romania; 6 Department of Dermatology, Elias Emergency University Hospital, 17 Bulevardul Marasti, 011461 Bucharest, Romania; catalin_enachescu@yahoo.com; 7 Department of Dental Medicine and Nursing, Faculty of Medicine, Lucian Blaga University of Sibiu, 550169 Sibiu, Romania; anca.fratila@ulbsibiu.ro; 8 Military Clinical Emergency Hospital of Sibiu, 550024 Sibiu, Romania

**Keywords:** magnetic nanoparticles, cellulose acetate, coating, biomineralization

## Abstract

Following implantation, infections, inflammatory reactions, corrosion, mismatches in the elastic modulus, stress shielding and excessive wear are the most frequent reasons for orthopedic implant failure. Natural polymer-based coatings showed especially good results in achieving better cell attachment, growth and tissue-implant integration, and it was found that the inclusions of nanosized fillers in the coating structure improves biomineralization and consequently implant osseointegration, as the nanoparticles represent calcium phosphate nucleation centers and lead to the deposition of highly organized hydroxyapatite crystallites on the implant surface. In this study, magnetic nanoparticles synthesized by the co-precipitation method were used for the preparation of cellulose acetate composite coatings through the phase-inversion method. The biomineralization ability of the membranes was tested through the Taguchi method, and it was found that nanostructured hydroxyapatite was formed at the surface of the composite membrane (with a higher organization degree and purity, and a Ca/P percentage closer to the one seen with stoichiometric hydroxyapatite, compared to the one deposited on neat cellulose acetate). The results obtained indicate a potential new application for magnetic nanoparticles in the field of orthopedics.

## 1. Introduction

The field of polymeric membranes is extensively studied due to the multiple practical applications owed to the remarkable properties of these materials, with the most important one being selectivity [1]. Although the main application for which these materials were developed was water filtration and purification [2,3], over time membranes were also developed for other applications, such as biomedical ones [4]. Membrane systems have been developed for the controlled release of drugs [5,6,7], for the separation and fractionation of proteins [8,9] or for the substitution of the function of various organs—including artificial oxygenators used during open-heart operations [10,11], artificial pancreases [12,13], or artificial livers [14,15]. Special attention is paid to hemodialysis membranes, used in patients with chronic kidney disease [16,17], with these membranes allowing for the patients to be kept alive by extracorporeal blood filtration once every two days. The use of membranes in a such large number of medical applications has led to comprehensive research directed towards the enhancement of their biocompatibility. Two different strategies have been approached in this direction—chemical modification of the membrane surface [18] or the use of natural polymer membranes [19]. The main natural polymers used to obtain membranes are starch, cellulose, pectin, gum, alginates, chitin and chitosan [20]. Out of all the polysaccharides used to obtain polymer membranes, the greatest applicability is attributed to cellulose derivatives. Cellulose, although it is the most abundant natural polymer on the planet, has the main disadvantage of being soluble only in mixtures of organic solvents, all of which have a very high toxicity [21,22]. Cellulose derivatives, on the other hand, in addition to their high chemical versatility given by the presence of hydroxyl groups, have the great advantage of being soluble in aprotic polar organic solvents [23,24].

In addition to the established applications of polymeric membranes in the aforementioned biomedical fields, in recent times research was mainly directed towards the design of membranes that favor osseointegration processes [25,26]. Such membranes are used especially as interface materials between the bone and the metal implant, having the role of favoring osseointegration by facilitating the proliferation of osteoblasts, thus welding the metal implant into the bone. One of the candidate polymers for this application is cellulose acetate, which, in addition to the chemical properties previously described (reactivity and solubility), is a bioresorbable polymer, degrading to glucose under physiological conditions in a process that can be accelerated and controlled by the acetylation degree of the polymer [27,28]. Recent studies showed that the functionalization of cellulose acetate membranes with crown ethers, followed by their activation with Ca^2+^ ions, led to good biomineralization properties on the membrane surface [29]. The use of hydroxyapatite and magnesium particles as fillers to obtain composite membranes also led to good results regarding the osseointegration capacity of magnesium implants [30].

Magnetic particles, obtained in nano or micro sizes, have a multitude of applications in the biomedical field, among which the most important ones are related to targeted drug delivery [31,32,33], medical imaging [34,35,36], osseointegration [37,38], or diagnostic sensors [39,40,41]. For example, composite polymer membranes based on cellulose acetate with magnetic particles have been synthesized by the mechanical despair of magnetic nanoparticles in a polymer solution, with the membranes being synthesized by solvent evaporation [42]. The compact films thus obtained were used for food packaging.

Polymeric nanocomposite biomaterials find many applications in cancer treatment as devices for the controlled release of cytostatic drugs [43], in soft tissue engineering [44] in bone regeneration [45] or in antibacterial purposes [46]. Nanocomposites for cancer treatment have the advantage of being able to incorporate functionalized nanoparticles into a polymer support matrix with the ability to release cytostatic drugs under the action of certain stimuli—including pH [47], temperature [48] or magnetic field [49]. In the field of osseointegration, the most common nanocomposites are based on hydroxyapatite nanoparticles embedded in various polymeric matrices such as poly(lactic acid), chitosan, cellulose derivatives. In addition to the osteoinductive function of generating bone and promoting the synthesis of hydroxyapatite around the scaffold or implant, hydroxyapatite-based nanocomposite biomaterials have the advantage of also having the ability to release active substances at the implantation site (especially antibiotics and anti-inflammatory drugs) [50]. Also, in the field of nanocomposite biomaterials for bone regeneration, species such as layered double hydroxide (LDH) have also been used as nanofillers—for controlled release of anti-inflammatories at the implantation site [51] or functionalized graphene—both for the osteoinductive effect and for the ability to release antisense oligonucleotides and to treat aggressive forms of bone cancer such as multiple myelomas [52]. In the field of bone regeneration, magnetic particles have the advantage of being excellent nucleation centers on which hydroxyapatite grows easily [53]. Moreover, it has been shown that hydroxyapatite grows around magnetic nanoparticles by engulfing them completely, with the newly formed hydroxyapatite having magnetic properties with obvious advantages in terms of cell proliferation and adhesion [54]. One of the main limitations of the use of magnetic nanoparticles in bone regeneration processes is related to their access path in the body and the support used [55].

In this article, we report for the first time the synthesis of a biocompatible and bioresorbable polymer composite membrane with magnetic nanoparticles to improve the biomineralization capacity (generation of hydroxyapatite on the membrane surface), using magnetic particles as nucleation centers. Also, the composite coatings were subjected to Taguchi biomineralization to analyze their biomineralization capacity in vitro. The neat cellulose acetate and the composite coatings were thoroughly characterized via FT-IR, XPS, XRD, SEM and EDS analysis before and after Taguchi biomineralization, and the results showed the successful integration of the nanoparticles in the membrane structure via molecular adsorption and an improved biomineralization ability of CA/Fe_3_O_4_ was reported compared to neat CA.

## 2. Materials and Methods

The purpose of this study was the synthesis of cellulose acetate/iron oxide nanoparticle composite membranes with improved biomineralization ability. Two main methods can be used for composite membrane synthesis—solution casting followed by solvent evaporation and phase inversion. In the case of composite membranes with magnetic nanoparticles, the most suitable one is phase inversion, due to the possibility of local agglomeration of magnetic nanoparticles inside the polymer solution film during solvent evaporation [56]. More than that, in the case of cellulose acetate membranes, this is also the most suitable synthesis method, with the membranes from this polymer obtained by solvent evaporation being more friable and losing their plasticity [57]. Therefore, both neat and composite CA membranes were prepared by phase inversion starting from a solution of cellulose acetate in N,N′-dimethylformamide (14 wt% concentration) and iron oxide nanoparticles in the case of the composite membranes. Based on previous results related to optimum ultrasonication time and optimum filler concentration in the membrane structure, a 1 wt% concentration of magnetic nanoparticles was dispersed in the polymer solution [58]. The nanoparticles were synthesized using a simple method of co-precipitation in alkaline environment, and they were incorporated in the polymer solution via ultrasonication to ensure a homogeneous dispersion of the filler throughout the composite membrane and avoid potential agglomeration. Detailed information about the materials used, nanoparticle synthesis and membrane preparation procedures are provided in the sections below.

### 2.1. Chemicals and Reagents

Cellulose acetate (CA) powder (average Mn~30,000 by GPC) was supplied by Sigma Aldrich (Burlington, MA, USA). DMF (99%, Honeywell, Charlotte, NC, USA) was used for polymer dissolution. Ferric chloride hexahydrate (FeCl_3_ * 6H_2_0), iron sulfate (Fe(SO_4_)_2_ * 6H_2_O) (Fluka, Buchs, Switzerland), sodium hydroxide and ammonium hydroxide (Merck, Rahway, NJ, USA) were used for the iron nanoparticle synthesis. Calcium chloride (94%, Roth, Karlsruhe, Germany), hydrochloric acid (37%, Sigma Aldrich, Burlington, MA, USA), tris(hydroxymethyl)aminomethane (99.8%, Sigma Aldrich, Burlington, MA, USA) and anhydrous disodium phosphate (99%, Sigma Aldrich, Burlington, MA, USA) were employed for the mineralization studies. All reagents were analytical grade and were used without further purification. The water utilized in all experiments was distilled water.

### 2.2. Synthesis of the Magnetic Nanoparticles

The magnetic nanoparticles were synthesized via the co-precipitation method in a strong alkaline environment [59]. First, ferric chloride and iron sulfate were dissolved in distilled water thus resulting Solution 1. Next, Solution 2 was prepared by mixing ammonium hydroxide and distilled water. Solution 1 was added drop-wise into Solution 2, at room temperature, under continuous magnetic stirring. The pH of the resulting solution was constantly maintained at 12 by sodium hydroxide addition. The black precipitate obtained after the complete drop-wise addition of Solution 1 into Solution 2 was separated using a 100 kgf magnet and purified via dialysis. The nanoparticles were thoroughly washed with distilled water, dried in a vacuum laboratory oven at 40 °C for 24 h and crushed using a ceramic mortar and pestle prior to characterization.

### 2.3. Preparation of the Composite CA/Fe_3_O_4_ Membranes

The composite membranes were prepared using a technique previously developed in our research group [60]. Briefly, the cellulose acetate solution (14% concentration) was obtained by dissolving the polymer powder in N,N′-dimethylformamide, under magnetic stirring at 600 rpm, for 6 h at 40 °C. After complete polymer dissolution, the iron oxide nanopowder was added (1 wt% with respect to the polymer mass) and the magnetic particles were homogeneously dispersed within the polymer matrix using an ultrasound ice bath (Ultrasonic processor UP100H, Hielscher Ultrasonics, Germany) at 80% amplitude for 25 min. Next, the membranes were cast on a glass plate using an Elcometer 4340 Automatic Film Applicator (Elcometer, Warren, MI, USA), and gently immersed in a coagulation bath containing 500 mL of distilled water at 25 °C. Finally, the membranes (surface area 10 × 10 cm, thickness 200 μm) were removed from the coagulation bath, thoroughly washed with ethanol and distilled water to remove the excess of DMF and dried in a vacuum laboratory oven, at 40 °C for 24 h, prior to characterization.

### 2.4. Biomineralization Study

The mineralization ability is a crucial feature of biomaterials designed for applications in osseointegration since numerous studies showed that mineralized biomaterials have improved bone regeneration enhancement ability compared to their non-mineralized counterparts. Preliminary studies performed to assess the mineralization ability in vitro are mainly based on chemical methods (e.g., simulated body fluid incubation, alternate soaking in calcium and phosphate ionic solutions) or biological methods such as mineralization by osteogenic cells [61]. Mineralization by cells is based on the ability of certain cells belonging to osteogenic lineages (e.g., mesenchymal stem cells) to naturally deposit minerals in their surrounding environment. However, the deposition parameters and nature of the deposited minerals are difficult to control and the technique requires specialized working conditions and prolonged time periods. Therefore, due to their accessibility, chemical techniques are applied more frequently [62]. The main chemical techniques that can be used to assess the mineralization ability of biomaterials in vitro are the simulated body fluid (SBF) or Kokubo technique and the alternate soaking or Taguchi method. These techniques were intensively applied in research studies and it was found that both of them are very effective in creating bone-like apatite layers on the material’s surface [63]. Although the SBF technique is straightforward when mild reaction conditions are used, and the chemical composition of the resultant apatite resembles that of natural bone more closely, the mineralization process is relatively slow and requires replenishing the solution at specific time intervals. High-concentration SBFs (such as 5 × SBF) can significantly speed up the biomimetic mineralization process; however, these solutions struggle to maintain a consistent pH level and are likely to form homogeneous calcium salts during the initial phases of biomimetic mineralization due to the release of gaseous CO_2_ [64]. Therefore, Taguchi et al. proposed a faster and more accessible procedure based on the widely known wet-process for hydroxyapatite preparation that consists of sequential mineralization via consecutive alternate immersion of the biomaterial in ionic solutions, which are typically calcium and phosphate-based. The deposition of Ca^2+^ and PO_4_^3−^ ions plays a crucial role in the mineralization of biomaterials for bone tissue engineering. However, a drawback of this technique is that the Ca/P ratio of the minerals formed largely relies on the sources of Ca^2+^ and PO_4_^3−^, as well as the concentration of each solution. Therefore, initial experiments must be conducted to fine-tune the conditions to achieve a Ca/P ratio that is close to the ideal value (1.67) [61].

In this research, the biomineralization studies were conducted using the alternate soaking method described by Taguchi et al. [65]. The samples were first incubated in a 200 mM CaCl_2_ solution at 37 °C for 24 h. The pH of the solution was adjusted to 7.4 using HCl and Tris base. Next, the membranes were briefly rinsed with distilled water and incubated for an additional 24 h in a 120 mM Na_2_HPO_4_ solution at 37 °C. The cycle was repeated two times. Finally, the membranes were rinsed with distilled water and dried for 72 h at 37 °C before characterization.

### 2.5. Characterization Methods

ATR FT-IR spectra were recorded using a Bruker VERTEX 70 spectrometer (Bruker, MA, USA), equipped with a diamond ATR device, in the 4000–600 cm^−1^ region, at 4 cm^−1^ resolution. The spectra were recorded as an average of 32 successive measurements for each sample.

The surface chemistry was studied by X-ray Photoelectron Spectroscopy (XPS) using a K-Alpha instrument from Thermo Scientific (Thermo Fisher Scientific, Waltham, MA, USA), with a monochromate Al Kα source (1486.6 eV), at a bass pressure of 2 × 10^−9^ mbar. Charging effects were compensated by a flood gun and binding energies were calibrated by placing the C1s peak at 284.4 eV as internal standard. A pass energy of 200 eV and 20 eV was used for survey and high-resolution spectra acquisition, respectively. The deconvolution of C1s, O1s and N1s spectra was done for both neat and functionalized CA using a Gaussian–Lorentzian function.

The morphology of the samples was visualized by scanning electron microscopy (SEM), using a Quanta Inspect F microscope (Hillsboro, OR, USA), equipped with an energy-dispersive X-ray spectrophotometer (EDX), with the accelerating voltage being set at 30 kV.

The phase composition and crystalline structure were investigated by X-ray diffraction using a Shimadzu XRD 6000 instrument (Shimadzu, Japan) with Cu Kα radiation (λ = 1.54 Å), filtered by Ni, with the 2θ angle being varied between 0 and 80°.

## 3. Results

To analyze their biomineralization capacity in vitro, 1 × 1 cm samples from the obtained membranes were subjected to Taguchi biomineralization and then characterized by FT-IR, XPS, XRD, SEM and EDS analysis. FT-IR EDS and XPS provided useful information regarding the chemical composition of the membrane surfaces while SEM images showed the initial membrane structure and the modifications that occurred following the nanoparticle addition and biomineralization. XRD spectra and EDS mapping revealed the nature of the deposited mineral compounds and highlighted the differences in the crystallinity and purity degree of the phosphates formed on the neat polymer membrane vs. the phosphates formed on the nanoparticle-containing one.

### 3.1. ATR FT-IR

**Figure 1:**
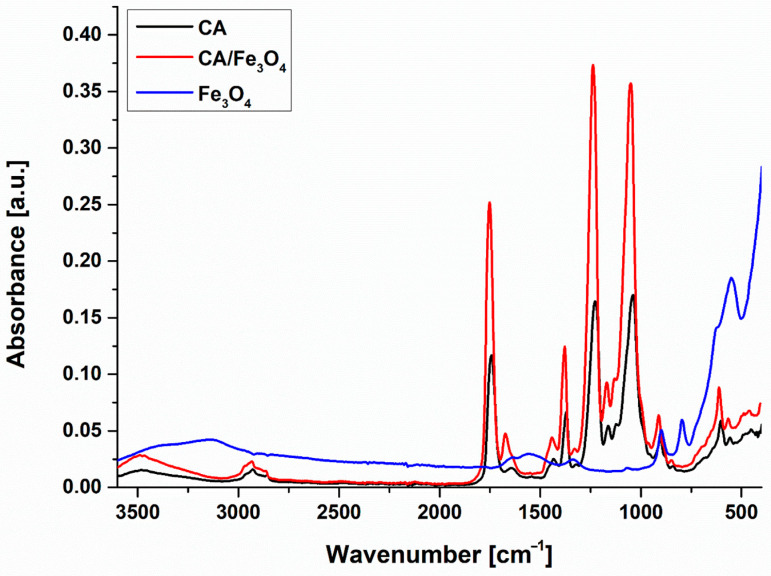
ATR FT-IR spectra of Fe_3_O_4_ nanopowder, neat CA membrane and CA/Fe_3_O_4_ composite membrane.

Figure 1 illustrates the ATR FT-IR spectra obtained for the Fe_3_O_4_ nanopowder, neat CA and CA/Fe_3_O_4_ composite membranes. The FT-IR spectrum of the nanopowder showed a well-defined peak at 550 cm^−1^, generated by the Fe-O stretching vibrations of the tetrahedral and octahedral sites of magnetite and two sharp peaks at 795 and 898 cm^−1^ that were attributed to the –OH stretching vibrations of goethite (α-FeOOH), a mineral consisting of iron oxide–hydroxide that can occur as a secondary product during magnetite nanoparticle synthesis [66,67].

A broad shoulder was also present in the 3500-3000 cm^−1^ area, indicating the presence of -OH functional groups on the nanoparticle surface [68]. The spectrum of the neat CA membrane was in good agreement with previously reported results, presenting the characteristic peaks for this polymer, more specifically at 1740, 1370 and 1225 cm^−1^, which can be attributed to the C=O, C–H and C–O stretching in the acetyl group, and at 1042, 902 cm^−1^, corresponding to the C1-H and O-H bending vibrations of the β-glycosidic linkages between the glucose molecules [29]. The adsorption bands resulted from the stretching vibrations of the –OH and C-H groups were also present at 3480, 2920 and 2850 cm^−1^, respectively [69]. No significant differences were noticed in the FT-IR spectrum of the CA/Fe_3_O_4_ composite membrane, most likely due to the low amount of nanopowder with respect to the polymer mass (1 wt%) and to the overlapping of the Fe-O adsorption bands with the ones that are characteristic of the fingerprint region of cellulose acetate. The presence of the nanoparticles in the membrane structure was however signaled by an intensity increase in the peak located at 1665 cm^−1^ that can be related to the bending vibrations of surface hydroxyl groups and may indicate a certain degree of molecular adsorption of the nanoparticles on the polymer backbone [66].

### 3.2. XPS

**Figure 2:**
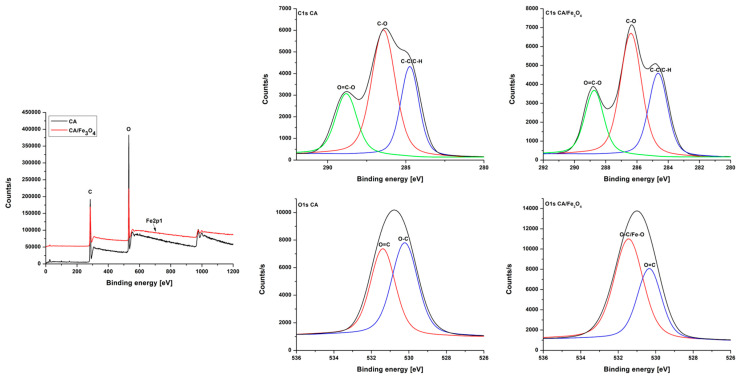
Survey and high-resolution C1s and O1s XPS spectra of neat CA membrane and CA/Fe_3_O_4_ composite membrane.

The XPS analysis (Figure 2) was performed to highlight the changes in the surface chemistry of the cellulose acetate membranes after the addition of the Fe_3_O_4_ nanopowder. The survey spectra of both neat CA and CA/Fe_3_O_4_ composite membranes showed two characteristic peaks, C1s and O1s, centered at 284.75 and 531.28 eV. A peak corresponding to Fe2p1 was also observed in the XPS spectra of the composite membranes at 722.05 eV, but, due to the low amount of iron detected by XPS (0.17%), the peak was small and not well defined. An explanation for this result is that XPS is a surface analysis technique and therefore it cannot detect iron-based nanoparticles located in the membrane structure, especially if the amount of filler is low with respect to the polymer mass. More conclusive results regarding the bulk chemical composition of the membranes were obtained via XRD assays. After deconvolution, the C1s spectra of the neat CA membrane presented with three sub-peaks situated at 288.83, 286.43 and 284.72 eV. These values were attributed to the O=C-O, C-O and C-C/C-H bonds, respectively [70]. No significant changes were noticed in the C1s spectrum of the composite membrane. The high-resolution O1s spectra of the neat CA membrane presented with two sub-peaks at 530.36 and 531.43 eV that were attributed to the O=C and O-C bonds in CA [70]. According to previous literature studies, the XPS signals corresponding to Fe-O bonds in the Fe_3_O_4_ structure generate a strong peak around the value of 531 eV in the high-resolution O1s spectrum [71,72]. In the case of the composite CA/Fe_3_O_4_ membranes, the presence of Fe_3_O_4_ nanoparticles was signaled by a visible intensity increase in the peak located at 531.44 eV in the high-resolution O1s spectrum that was attributed to the contribution of Fe-O bonds from the Fe_3_O_4_ structure.

### 3.3. SEM

**Figure 3:**
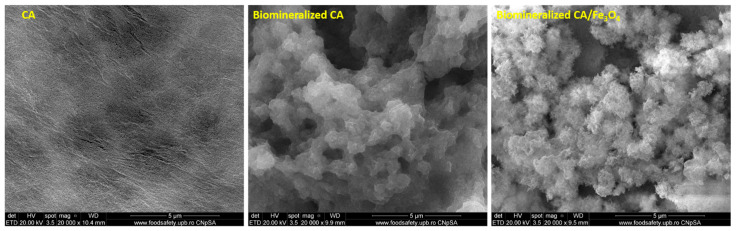
SEM micrographs of neat cellulose acetate membrane, biomineralized cellulose acetate and biomineralized CA/Fe_3_O_4_ composite membrane.

The membranes in this study were obtained using the phase inversion method, which consists of polymer dissolution in an appropriate solvent (in this case, N,N′-dimethylformamide) to obtain a homogeneous solution that is cast on a glass plate and submerged in a coagulation bath containing a non-solvent, usually distilled water. Demixing and polymer precipitation result from the solvent’s penetration into the non-solvent and the non-solvent’s penetration into the polymeric solution. This creates an asymmetric membrane that is typically made up of a thin film top layer, also known as “skin”, with a support porous substructure as the bottom layer [73]. The SEM micrographs (Figure 3) of the neat CA membranes before mineralization show the structure described above, with a thin, dense active layer at the surface and a porous substrate below, characterized by homogeneous rounded pores, with diameters around 3 μm. This hierarchical organization was no longer observed after the biomineralization process, neither in neat CA nor in composite CA/Fe_3_O_4_ membranes due to the large quantity of mineral deposition on the membrane surface. The minerals were evenly disposed across the entire analyzed area forming grape-like clusters of micrometric dimensions comprised spherical calcium phosphate particles. On the surface of the composite membranes, the calcium phosphate crystals were needle-shaped and highly organized into flower-like structures. This nanoparticle-mediated organization of biomimetically deposited minerals was also observed by Liu et al. during their studies regarding the biomimetic mineralization of magnetic iron oxide nanoparticles. It was concluded that, due to their high surface area, reactivity, shape and size, inorganic nanoparticles could represent nucleation sites and provide a favorable template for the formation and growth of calcium phosphate crystals during biomimetic mineralization [74]. Other studies also reported that hydroxyl-rich surfaces greatly enhance the nucleation and growth of calcium phosphates via a one-step nucleation process [75], therefore, the -OH functional moieties that were, according to FT-IR analysis, present on the nanoparticle surface, could also be an explanation for the higher amount and degree of organization in the minerals deposited on the composite membranes compared to the neat ones.

### 3.4. EDS

**Figure 4:**
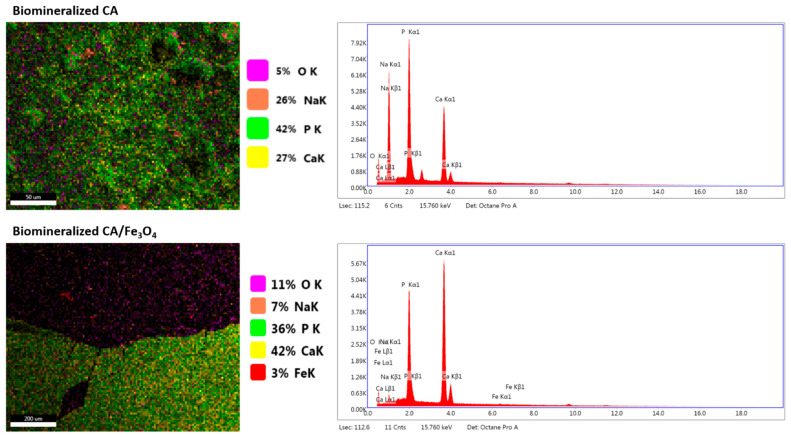
EDS elemental maps, percentages and spectra of neat and CA/Fe_3_O_4_ composite membranes after Taguchi biomineralization.

EDS analysis (Figure 4) was performed in order to analyze the chemical composition of the neat and composite membrane surfaces after the Taguchi biomineralization process. The surface elemental composition of the neat biomineralized membrane primarily comprised phosphorus, calcium and sodium. Small percentages of oxygen were also present. The high density of the mineral layer was highlighted by the fact that carbon was not visible in the elemental map due to the complete coverage of the polymeric substrate with calcium phosphates during biomineralization. Moving on to the composite membrane, the first observation that can be made is that the presence of the iron oxide nanoparticles not only led to the formation of highly organized, well-defined calcium phosphates crystals on the membranes surface, as revealed by SEM micrographs, but also contributed to the obtainment of minerals with a higher degree of purity, this being suggested by the higher percentages of calcium, phosphorus and oxygen and lower sodium percentages. Moreover, the Ca/P percentages calculated directly from the EDS results showed that the value obtained for the phosphates deposited on the composite membranes surface was higher and therefore closer to the one of stoichiometric hydroxyapatite (1.16 vs. 0.62 for the neat membranes) [76]. Considering these results, it can be concluded that the nature of the calcium phosphates deposited on the composite membranes can be related to calcium deficient hydroxyapatite while the ones on the neat membranes are most likely a mixture of amorphous calcium and sodium phosphates [77].

### 3.5. XRD

**Figure 5:**
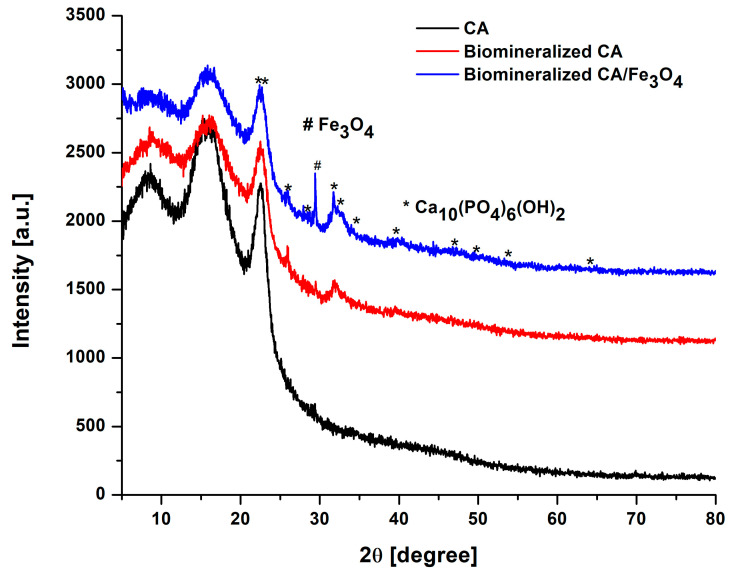
XRD spectra of neat and CA/Fe_3_O_4_ composite membranes before and after Taguchi biomineralization.

Figure 5 illustrates the XRD spectra of the neat and composite cellulose acetate membranes before and after Taguchi biomineralization. The main peaks obtained for both neat CA and biomineralized CA and CA/Fe_3_O_4_ were present at the approximate values of 2θ = 8.91°, 15.99° and 22.56°. These peaks were attributed to the crystalline planes 010 from cellulose III, and 110 and 200 from cellulose I [78,79]. Along the three well-defined peaks belonging to cellulose acetate [80], several signals corresponding to hydroxyapatite were distinguished in the spectra of the biomineralized membranes between 2θ = 25.91° and 64.05° [81,82] and, in the case of the composite membrane, the presence of Fe_3_O_4_ nanoparticles was confirmed by a strong peak at 2θ = 29.42°, belonging to the crystalline plane 220 in the magnetite structure [83]. Therefore, the presence of calcium phosphates in the form of hydroxyapatite was confirmed in the membrane structure. The higher intensity of the peaks belonging to hydroxyapatite in the composite membrane spectrum suggests that the biomineralization process was promoted by the presence of Fe_3_O_4_ nanoparticles and, in correlation with the SEM and EDS results, it can also be stated that the purity and crystallinity of the obtained calcium phosphate were also enhanced.

## 4. Discussion

Bone regeneration materials [84,85,86,87,88] represent one of the most important directions in the field of biomaterials, primarily because of their necessity and immediate applicability on a large scale—especially in dentistry and orthopedics. In addition to the physicochemical characteristics that need to be precisely determined [89], the biological testing of these materials is decisive in determining whether they can be used for the biomedical application for which they were designed [90]. One of the easiest methods of characterization is biomineralization ability assessment. Biomineralization is the ability of the synthesized material to gain hydroxyapatite at its surface based on physicochemical processes that would facilitate the formation of hydroxyapatite crystals [91]. In addition to this ability, the material must also be bioresorbable over time to make room for the newly formed hydroxyapatite or bone tissue [92]. Out of all natural polymers, polysaccharides have the advantage of degrading under physiological conditions forming glucose or other simple saccharides, thus the impact on the body being minimal. Without using surface functionalization molecules or a filler for the synthesized material, their ability to generate hydroxyapatite at the surface is minimal. This ability can be induced and improved either by immobilizing molecules on the surface that complex Ca^2+^ ions, thus retaining them on the surface, or by adding fillers that form nucleation centers that allow for crystal growth. Here, we report a new composite membrane material based on cellulose acetate and magnetic nanoparticles. The ability of hydroxyapatite generation is given by the adsorption of Ca^2+^ ions at the surface of magnetic nanoparticles, which represent nucleation and growth centers for hydroxyapatite crystals. A comparison with previously reported cellulose and cellulose derivative-based materials membranes for biomineralization is made in Table 1.

As can be seen from the data presented in Table 1, the use of cellulose leads to the formation of a mixture of crystals such as aragonite, brushite and hydroxyapatite [94] or octacalcium dihydrogen hexakis phosphate (V) pentahydrate [95], with this being due to the low ability of this polymer to retain Ca^2+^ ions on the surface, regardless of its shape. This capacity is given only by the free electrons of the oxygen atoms in the cellulose component. In the case of carboxymethyl cellulose, where carboxyl groups can easily complex Ca^2+^ ions, hydroxyapatite crystals with a Ca/P ratio close to the ideal one are obtained [93]. The same results were obtained in the case of cellulose acetate where the complexing capacity of the C=O group is higher than that of -OH [93]. In the case of crown ether functionalization of cellulose acetate [29], their high complexing capacity facilitated the synthesis of a large amount of hydroxyapatite on the membrane surface. The magnetic particles, which favor the fixation of calcium ions to their surface by adsorption [96], also facilitated the synthesis of hydroxyapatite, with the lower Ca/P ratio than in the case of natural hydroxyapatite being explained by the agglomeration of Ca^2+^ ions around the magnetic particles and implicitly by a steric impediment around the nucleation centers.

The use of magnetic nanoparticles for biomedical applications is well known, especially in the treatment of various forms of cancer [97]. They can be used in combination with thermal therapy [98] or as a carrier for the various active substances [99]. For applications involving the circulation of these nanoparticles in the blood, the parameter that best controls the toxic or nontoxic characteristics is the size of the nanoparticles. Well dispersed particles in nanometric dimensions can be easily eliminated (either through the kidneys or with proteins complexed in fecal matter) after performing the therapeutic function [100]. In the case of magnetic nanoparticles used for biomineralization processes, they remain locked in the newly formed mineral structure, with multiple studies showing that hydroxyapatite with magnetic properties has superior properties in terms of cell adhesion and biocompatibility [101,102].

## 5. Conclusions

The purpose of this study was the preparation of composite cellulose acetate/iron oxide nanoparticles with applications as coatings for metallic implants in order to promote and improve their biomineralization. The iron oxide nanoparticles were synthesized via co-precipitation in a strong alkaline environment and dispersed within the polymer matrix using an ultrasound ice bath. Their presence in the composite membrane structure was signaled by an intensity increase in the FT-IR peak located at 1665 cm^−1^, corresponding to the bending vibrations of surface hydroxyl groups that indicated the molecular adsorption of the nanoparticles on the polymer chains. Also, the XPS, EDS and XRD spectra showed characteristic signals for magnetite in the case of the composite membranes. More specifically, a visible intensity increase in the peak located at 531.44 eV in the high-resolution O1s XPS spectrum was observed that was attributed to the contribution of Fe-O bonds, as well as several iron-specific signals (Fe Lβ1, Fe Lα1, Fe Kβ1, Fe Kα1) in the EDS elemental spectrum, and a strong peak at 2θ = 29.42°, belonging to the crystalline plane 220 in the magnetite structure in the XRD spectrum. The characterization results also highlighted the fact that the presence of the iron oxide nanoparticles promoted biomineralization, with higher quantities of calcium phosphates being deposited on the composite membrane surface compared to the neat ones. More than that, due to their unique features (e.g., shape, size, surface energy, surface functional groups, etc.) the nanoparticles led to the formation of calcium phosphates with a higher purity degree and Ca/P percentage closer to those of stoichiometric hydroxyapatite. Future trends include coating titanium-based materials with the developed cellulose acetate/iron oxide nanoparticle membranes and studying their physico-chemical and biological characteristics in comparison with neat titanium.

The limitations of the proposed material for industrial or clinical applications are especially related to the synthesis methods. The obtainment of magnetic nanoparticles for biomedical applications at an industrial scale is very expensive due to the fact that homogeneity in terms of size and shape must be assured. Also, there is an issue with the synthesis of composite membranes in general, with the dispersion of nanofillers at industrial scale for preparing large quantities of membranes being currently impossible.

## Figures and Tables

**Table 1:** Comparison between different cellulose and cellulose derivative-based membranes and biomineralization tests results.

Type of Polymer	Membrane Synthesis Method	Mineralization Procedure	Mineralization Results	References
Cellulose acetate functionalized with activated crown ethers	Phase inversion for membrane formation followed by covalent immobilization of crown ethers	Taguchi biomineralization/incubation in Simulated Body Fluid (SBF)	Hydroxyapatite synthesis at surface of functionalized membrane	[29]
Cellulose acetate	Electrospun fiber-based membrane	Taguchi biomineralization/incubation in Simulated Body Fluid (SBF)	Crystal formation at surface of membrane with Ca/P ratio similar to hydroxyapatite in natural bone	[93]
Cellulose	Commercial dialysis membrane	Incubation with biological fluids from freshwater bivalve Anodonta cygnea	Crystal formation at membrane surface with a mixture of aragonite, brushite and hydroxyapatite	[94]
Bacterial nanocellulose	Membrane obtained by bacterial nanocellulose fermentation with *Komagataeibacter medellinensis*	Five alternative soaking cycles in calcium and phosphate salts solutions	Main mineral obtained at membrane surface was octacalcium dihydrogen hexakis phosphate (V) pentahydrate	[95]
Carboxymethyl cellulose	Nonwoven sheets obtained from cotton linter	Alternate soaking in aqueous solutions of CaCl_2_/Tris-HCl and NaH_2_PO4	Crystal formation at the surface of membrane with Ca/P ratio similar to hydroxyapatite in natural bone	[96]

## Data Availability

The raw data are available from the corresponding author by request.
